# Simultaneous Autophagy and Androgen Receptor Inhibition in a Prostate Cancer Xenograft Model

**DOI:** 10.3390/cancers16193261

**Published:** 2024-09-25

**Authors:** Souzan Salemi, Benedikt Kranzbühler, Valentin Baumgartner, Lara Breitenmoser, Aleksandar Kuzmanov, Fabienne Lehner, Daniel Eberli

**Affiliations:** Laboratory for Urologic Oncology and Stem Cell Therapy, Department of Urology, University Hospital Zürich, Wagistrasse 21, 4.OG, 8952 Schlieren, Switzerland; benedikt.kranzbuehler@usz.ch (B.K.); valentin.baumgartner@usz.ch (V.B.); fabienne.lehner@usz.ch (F.L.); daniel.eberli@usz.ch (D.E.)

**Keywords:** autophagy, prostate cancer, abiraterone, in vivo, PSMA

## Abstract

**Simple Summary:**

Recent advancements in anticancer drug treatments face significant challenges due to drug resistance, often leading to only partial or short-term responses in patients. Our previous in vitro studies indicated that autophagy is linked to drug resistance in prostate cancer (PCa). Consequently, combination therapies targeting multiple pathways could help overcome resistance and extend treatment efficacy. To validate our earlier findings, we conducted in vivo experiments. Our data demonstrated that combining Abiraterone (Abi) with an autophagy inhibitor such as Chloroquine (Chl) not only reduces autophagy but also suppresses tumors more effectively than Abi alone. Thus, the combination of Abi with autophagy inhibitors represents a promising therapeutic strategy that could potentially offer a novel approach for patients.

**Abstract:**

Objective: Abi, when used in conjunction with prednisone, is an established treatment for advanced PCa. Our goal was to explore the level of autophagy induced by Abi treatment, both alone and in combination with the autophagy inhibitor Chl, in a castrated mouse xenograft model. Methods: LNCaP cells were injected into the left and right sides of the back of nude mice that had been previously castrated. Mice were divided into four groups and treated daily with intraperitoneal injections of vehicle (control), Abi (10 mg/kg), Abi (10 mg/kg) combined with Chl (10 mg/kg), or Chl (10 mg/kg), and were monitored for periods of 2 and 3 weeks. Results: A significant reduction in tumor weight was observed in mice treated with the combination therapy, as opposed to those receiving vehicle control, Abi, or Chl alone. Mice receiving Abi + Chl exhibited reduced expression of ATG5, Beclin 1, and LC3 punctuations, along with an increase in P62, as determined by immunofluorescence and WES analysis. AR expression decreased significantly in all treatment groups compared to the control. PSMA expression was highest in the vehicle and combined treatment groups after 3 weeks, with a significant reduction observed with Chl treatment. Conclusions: These findings demonstrate that Abi + Chl treatment lowers autophagy levels and suppresses tumors more effectively than Abi alone.

## 1. Introduction

Blocking the androgen axis has been a standard treatment for advanced prostate cancer in men for several years. However, a majority of these patients eventually develop castration-resistant prostate cancer (CRPC) with metastases, particularly in the bone [1]. To block the androgen receptor (AR) in patients with advanced prostate cancer, current guidelines suggest a combined therapy with second-generation antiandrogens such as abiraterone, apalutamide, enzalutamide, or darolutamide [2,3]. To overcome the resistance observed with current therapies, the search for new AR-targeting compounds or combination therapies continues. Abiraterone acetate (Abi), a prodrug of abiraterone, robustly inhibits 17-hydroxylase/C17 (CYP17), a crucial enzyme in testosterone production [4]. This inhibition effectively suppresses androgen production in testicular, adrenal, and prostatic tumors [5]. In randomized prospective clinical trials, the combination of Abi and prednisone represents the first endocrine therapy demonstrating a significant survival advantage for patients with CRPC [6]. However, similar to other antitumor agents, some prostate cancer patients eventually develop resistance to Abi, while others may not respond to it at all [6].

Autophagy is a fundamental process in cells that facilitates self-degradation of cellular components, thus playing a key role in maintaining cellular homeostasis [7]. Numerous research groups, including our own, have provided evidence of dysregulated autophagy in prostate cancer [8,9,10]. Our prior investigations have revealed that inhibiting autophagy in cells treated with anticancer drugs such as EPI-001, Abi, and apalutamide, either through pharmacological means or via siRNA, results in increased cell death and enhances the effectiveness of anticancer drugs [8,11]. Furthermore, we have demonstrated that proteins associated with autophagy are significantly upregulated in patients with advanced prostate cancer [12]. Considering the encouraging antitumor effect of Abi in the treatment of advanced prostate cancer and the involvement of autophagy as a mechanism of resistance to therapy [10,11], we hypothesize that prostate cancer cells utilize autophagy as a survival strategy to evade the stress caused by androgen deprivation or antiandrogen therapies.

Therefore, our objective was to examine the extent of autophagy in response to Abi treatment alone and in combination with the autophagy inhibitor Chloroquine (Chl), while also exploring its correlation with prostate-specific membrane antigen (PSMA) and the AR axis in a castrated mouse xenograft model.

## 2. Materials and Methods

### 2.1. Cell Culture

The prostate cancer cell line LNCaP (ATCC, CRL-1740, Manassas, VA, USA) was obtained from LGC Standards GmbH (Wesel, Germany). Cells were cultured in RPMI medium (Life Technologies, ThermoFisher SCIENTIFIC, Waltham, MA, USA) supplemented with 10% FBS and 1% penicillin/streptomycin, and incubated at 37 °C with 5% CO_2_. The medium was replaced twice a week.

### 2.2. Animal Experimentation

All experiments involving animals were conducted in compliance with the Swiss Animal Welfare Act and approved by the cantonal veterinary office (Veterinäramt Zürich, Licence No. 244/2016, Zürich, Switzerland). A total of 28 male nude mice (8 weeks old; Charles River Laboratories, Sulzfeld, Germany) were studied. All mice were castrated prior to tumor formation [13]. After a 2-week interval, nude mice received subcutaneous injections of 5.0 × 10^6^ LNCaP cells suspended in a high-concentration Matrigel carrier (500 µL, Corning Life Sciences, New York, NY, USA) on both the left and right sides of their backs. The administration of the drug injection commenced once the tumors had developed, approximately 2 weeks subsequent to the injection of tumor cells. The mice were divided into 4 groups (3 animals/group/time point). Administration of the drug via intraperitoneal injections (i.p.) started three weeks after the initial tumor cell injection, once tumors had formed. The treatment groups vehicle control (0.037 N HCL, 20% 2-hydroxypropyl-betas-cyclodextrin, 0.13× phosphate buffer saline, purchased from Sigma Aldrich, Buchs, Switzerland), Abi (10 mg/kg, Janssen Pharmaceutica NV, Beerse, Belgium), Abi (10 mg/kg) + Chl (10 mg/kg, Sigma Aldrich, Buchs, Switzerland), and Chl (10 mg/kg) were subjected to intraperitoneal (i.p.) injections 5 days a week for a duration of 2 or 3 weeks. The mice did not meet the termination criteria. Caliper measurements were performed following the start of treatments and at the end of the experiment. At the end of the experiments, the animals were sacrificed, and all samples were assessed for tumor weight and size.

### 2.3. Processing and Histological Assessment of Tumor Samples

The tumor samples obtained from each mouse were divided into two parts: one was quickly frozen for gene and protein analysis, while the other was fixed in 4% buffered formaldehyde, processed, and embedded in paraffin (Sargent-Welch Scientific, Skokie, IL, USA). Paraffin sections, cut to a thickness of 5 μm, were then stained with Haematoxylin and eosin (H&E, Sigma Aldrich, Buchs, Switzerland) following the manufacturer’s guidelines.

### 2.4. Immunofluorescent Staining

The tissue sections were subjected to immunostaining with primary antibodies as follows: anti-ATG5 (1:100, 0262-100, 7C6, nanoTools, Taningen, Germany), anti-LC3 (1:100, 0231-100, 5F10, nanoTools, Taningen, Germany), anti-P62 (1:50, NB P1-48320, Novus Biologicals Europe, Abingdon, UK), anti-PSMA/FOLH1 (1:100, clone 460 420, R&D Systems, Zug, Switzerland), anti-caspase 3, active (1:100, cleaved, AB3623, Merck, Lucerne, Switzerland), and anti-Ki67 (1:100, AB9260, Merck, Switzerland). The secondary antibodies used, goat anti-mouse FITC (1:500, BD Biosciences Allschwil, Switzerland), goat anti-rabbit FITC (1:500, Vector Laboratories, Liestal, Switzerland), or Cy3-conjugated goat anti-mouse antibody (1:500, Sigma Aldrich, Buchs, Switzerland), were counter-stained with DAPI (4′,6-diamidino-2-phenylindole, Sigma Aldrich, Buchs, Switzerland). For negative controls, the primary antibody was left out. Images were captured using a Leica fluorescence microscope (Leica, CTR 6000, Wetzlar, Germany). Image quantification was performed using Image J (1.54 f NIH, Bethesda, MD, USA).

### 2.5. Simple Western (Automated Western Blotting—WES)

Crushed tumor samples in liquid nitrogen were resuspended in lysis buffer with a protease inhibitor cocktail (Sigma-Aldrich, Buchs, Switzerland). Protein concentration was measured using a BCA protein assay kit (Thermo scientific, Lausanne, Switzerland). A 1 mg/mL protein concentration was utilized for the WES using a 12–230 kDa cartridge kit (Protein Simple WES, Wiesbaden, Germany).

Primary antibodies targeting autophagy included anti-ATG5 (1:100, 0262-100, 7C6, nanoTools, Taningen, Germany), anti-Beclin 1 (1:50, NB110-87318, Novus Biologicals Europe, Abingdon, UK), anti-P62 (1:50, NB P1-48320, Novus Biologicals Europe), anti-PSMA/FOLH1 (1:100, clone 460420, R&D Systems, Minneapolis, MN, USA), and AR (1:500, clone D6F11, CellSignaling, Leiden, The Netherlands). Anti-glyceraldehyde 3-phosphate dehydrogenase (GAPDH 1:100, NB 300-221, Novus Biologicals Europe, Abingdon, UK) was used as an internal control. The samples were analyzed with the Compass software (ProteinSimple, 6.1.0, San Jose, CA, USA). Both virtual blots and electropherograms for each sample were examined and evaluated. The Compass software measured the clearly defined chemiluminescent signals and normalized the area of each sample to GAPDH.

### 2.6. Statistics

Results were analyzed using one-way ANOVA with Tukey’s post hoc test, performed with GraphPad Prism (GraphPad Software, Inc., La Jolla, CA, USA, version 7). *P*-values less than 0.05 were considered statistically significant. Data are presented as means with the standard error of the mean (±SEM).

## 3. Results

The combination of Abi and an autophagy inhibitor was effective in suppressing tumor growth in a mouse xenograft model.

**Figure 1:**
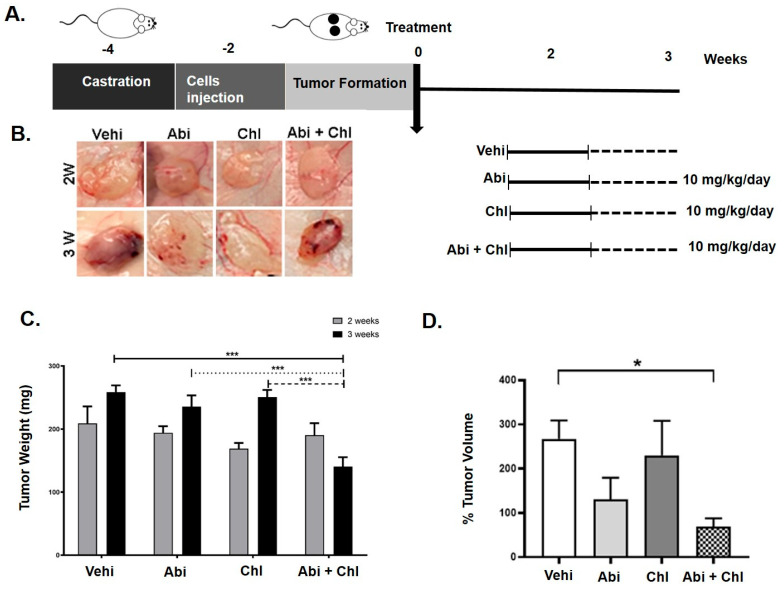
Impact of abiraterone (Abi), hydroxyl chloroquine (Chl), and their combination in a xenograft mouse model. (**A**) Diagram illustrating the animal study design and schedule of injections. (**B**) In vivo tumor growth was assessed in a mouse xenograft model treated with Abi alone, Chl alone, and their combination. LNCaP prostate cancer cells were subcutaneously injected into both sides of the backs of castrated nude mice and allowed to grow for 2 weeks. Mice were treated 5 days per week for 2 and 3 weeks with vehicle control, Abi (10 mg/kg), Chl (10 mg/kg), or both in combination (Abi 10 mg/kg + Chl 10 mg/kg). (**C**) Total tumor weight following tissue harvesting after 2 and 3 weeks upon treatments (N = 6) per experimental condition. (**D**) Caliper measurements (tumor volume) presented as a percentage of volume after 3 weeks to initial size (N = 6). Statistical significance is shown by asterisks: * *p* < 0.05; *** *p* < 0.0005.

In order to validate our earlier in vitro results [9] and assess the effects of a combined treatment involving Abi plus an autophagy inhibitor, we created a xenograft model of prostate cancer. This model involved the implantation of LNCaP cells into castrated male athymic nude mice, allowing us to analyze the in vivo growth dynamics (Figure 1A). All injected mice developed palpable tumors (Figure 1B).

Our findings indicated pronounced tumor growth in the control group 2 and 3 weeks after cell injection (Figure 1C). A decrease in tumor weight was noted in all experimental groups compared to the vehicle control at both weeks 2 and 3. Following 3 weeks of treatment, mice receiving Abi + Chl demonstrated the most considerable reduction in tumor weight, measuring 140.4 ± 14.0 mg (SEM, *p* = 0.0005), which was significantly lower than the tumor weights observed in mice treated with vehicle control (258.7 ± 10.6), Abi (235.7 ± 17.7), or Chl (250.6 ± 11.42) alone (Figure 1C).

Consistent with the weight results, caliper measurements revealed a significant reduction in tumor volume in all treatment groups compared to the untreated vehicle control (Figure 1D). Throughout the study, the animals’ mean body weight was stable and did not significantly vary between the treatment groups. No redness, swelling, or behavioral changes were observed in the Abi, Chl, or Abi + Chl treated animals, indicating no signs of stress or infection relative to the vehicle control.

**Figure 2:**
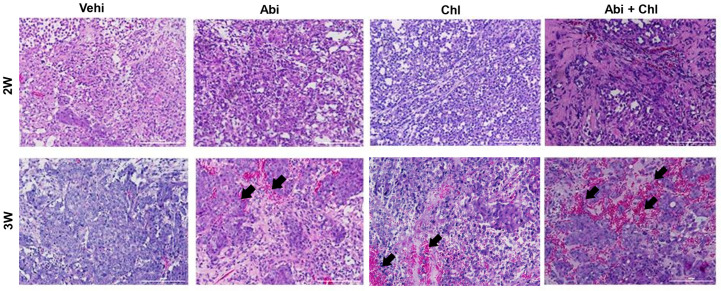
Sample images of paraffin-fixed tumor sections stained with hematoxylin and eosin under all experimental conditions are shown after 2 and 3 weeks of treatment with Abi, Chl, and their combination. Hematoxylin stained the nuclei blue–purple, while eosin stained the cytoplasm and red blood cells pink. Tumor tissue sections from animals treated with the combination of Abi + Chl showed increased infiltration of inflammatory cells and reduced cell population density. Black arrows indicate the increase in inflammatory cells. Scale bar: 200 µm.

The results indicate that the Abi + Chl treatment was well tolerated by the mice, and the dosages did not lead to any significant adverse reactions throughout the experiment. Confirmation of tumor formation was achieved through H&E staining of samples from various experimental conditions. After 3 weeks, all treatment groups showed elevated inflammation and increased nucleation compared to the vehicle control group (Figure 2).

### 3.1. Impact of Abi Treatment on Autophagy-Related Proteins

**Figure 3:**
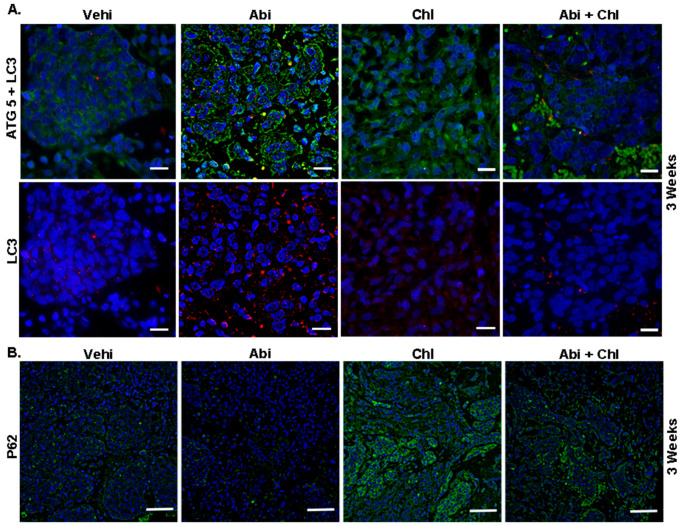
Upregulation of ATG5 and LC3 in Abi-treated animals. Representative immunofluorescent staining of tumor sections from all experimental conditions after 3 weeks of treatment is shown. (**A**) The green color indicates cytoplasmic expression of ATG5, while the red color indicates LC3 staining, representing autophagosome formation. Upregulation of ATG5 and LC3 punctuation depicts high autophagic activity in Abi-treated animal tissue sections. Tumor tissue sections from combination-treated animals showed decreased ATG5 expression. Scale bar: 20 µm. (**B**) The influence of treatments on P62 protein expression is demonstrated. Increased P62 (green) expression is observed in Chl and Abi + Chl-treated animals compared to Abi-treated animals. Samples were stained using a Cy3 (red) conjugated secondary antibody, FITC (green), and DAPI (blue, 4′,6-diamidino-2-phenylindole). Scale bar: 100 µm.

We investigated the expression of key autophagy proteins ATG5, LC3, and P62 in tumor tissue to evaluate potential Abi and autophagy inhibitors for future PCa treatment. The vehicle control exhibited minimal basal expression levels of ATG5 and faint, diffuse staining of LC3, as revealed by immunofluorescence (Figure 3). After 3 weeks of treatment, mice treated with Abi demonstrated elevated ATG5 expression and a punctuated LC3 pattern, confirming the buildup of autophagosomes (Figure S1). Mice receiving the combined Abi and Chl treatment exhibited reduced ATG5 expression and minimal LC3 punctuations (Figure 3, upper panel).

**Figure 4:**
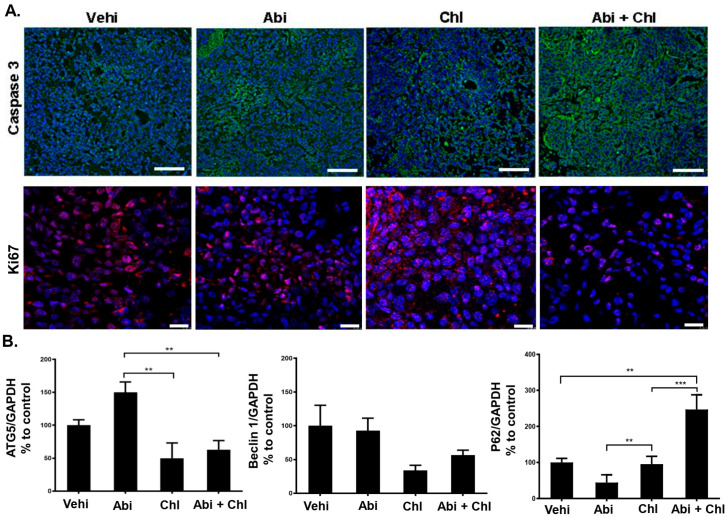
Influence of treatments on caspase 3 and Ki-67. (**A**) Upper panel: cytoplasmic staining of cleaved (active) caspase 3 (green) in tumor sections after 3 weeks of treatments. Lower panel: Ki-67 (red) immunostaining shown in the nuclei of tumor cells and cleaved (active) caspase 3 in the cytoplasm of tissue sections were measured and compared to vehicle control (set to 100%). Nuclei were stained with DAPI (blue). Scale bar: upper panel: 100 µm; lower panel: 20 µm. (**B**) WES quantification of ATG5 (N = 6), Beclin 1 (N = 6), and P62 (N = 5). The protein expression in each sample was normalized to its own GAPDH and analyzed using the Compass software (ProteinSimple). Statistical significance is shown by asterisks: ** *p* < 0.001; *** *p* < 0.0002.

Furthermore, reduced autophagy was correlated to an increase in P62 in the Chl alone and the combination treatment groups (Figure 3, lower panel). In mice treated with Abi + Chl, there was a notable increase in caspase 3 expression compared to the vehicle control. Therefore, in the combination group, inhibition of autophagy by Chl + Abi led to inducing apoptosis and the activation of cleaved caspase 3 (Figure 4A and Figure S2). Moreover, animals treated with Abi alone or Chl alone also displayed increased apoptosis compared to the vehicle control. Ki-67 nuclear staining was present in all samples, but was diminished in Abi + Chl-treated specimens (Figure 4A, lower panel). Moreover, the involvement of autophagy was also validated at the protein level through quantitative automated immunoblotting. Mice treated with Abi displayed elevated levels of ATG5 (143.1 ± 16.3, SEM), a key regulator of autophagy, compared to the vehicle control group (100%) (Figure 4B and Figure S3). A decrease in the expression of ATG5 was observed following treatment with autophagy inhibitor Chl (50 ± 23.1) and Abi + Chl (62.9 ± 93). Animals treated with Abi did not exhibit any changes in the expressions of Beclin 1 (92.6 ± 18.5). However, treatment with Chl (34.04 ± 7.3) alone and Abi + Chl (56.6 ± 7.2) resulted in a notable reduction of Beclin 1 compared to the vehicle control. The increase in autophagy led to a corresponding reduction in the expression of ubiquitin-binding protein P62 (20.22 ± 8.0) in Abi-treated animals compared to the vehicle control group. Inhibition of autophagy and dual treatment resulted in a significant increase in P62 protein expression in Abi-treated animals and a significant increase in the Abi + Chl combination group.

### 3.2. AR and PSMA Expression Following Treatment with Abi, Chl, and Their Combination

**Figure 5:**
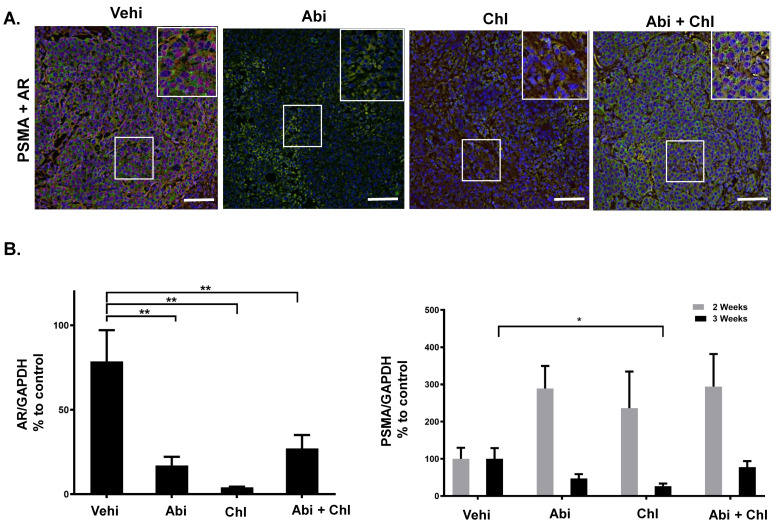
PSMA and AR expression in tumor tissues. (**A**) Visualization of PSMA and AR using immunofluorescent staining of tumor sections from all experimental conditions after 3 weeks of treatments: PSMA: green, FITC; AR: red, Cy3. The areas outlined in white boxes were enlarged in the upper right corner. All samples were counter-stained with DAPI. (**B**) Protein simple WES. Percentage of AR (N = 6) and total PSMA (N = 6) expression in tumor tissues from all experimental conditions. Expression of each sample was normalized to GAPDH. Data are shown as means with standard error of the mean (±SEM). Asterisks indicate statistically significant differences: * *p* < 0.05; ** *p* < 0.001. Scale bar: 100 µm.

To assess the expression of AR and PSMA in tumor tissue following treatment with Abi, Chl, and their combination, we performed immunofluorescent staining and quantitative automated immunoblotting. The presence of AR was exclusively observed in the vehicle control group and notably decreased in mice treated with Abi, Chl, and Abi + Chl, as visualized by both techniques (Figure 5A,B and Figure S4). Immunofluorescent staining revealed the expression of PSMA in all experimental groups, with the highest expression observed in the vehicle and double treatment groups after 3 weeks of treatment (Figure 5A). The changes in PSMA expression were assessed at the protein level by quantitative immunoblotting. Interestingly, there was an increase in PSMA protein expression after 2 weeks in response to all the treatments. However, after 3 weeks of treatment, PSMA expression decreased in all treatment groups, with significant reduction observed particularly in Chl treatment (Figure 5B and Figure S4).

## 4. Discussion

Acknowledging the significance of androgen receptor signaling as critical targets in prostate cancer treatment and recognizing the constraints imposed by autophagy on the efficacy of antiandrogen therapy, our study sought to determine whether modulating autophagy could amplify the effectiveness of antiandrogen therapy with Abi in an in vivo mouse model. The investigation also examined the link between AR and PSMA in relation to autophagy. The present study validates our in vitro findings [9], demonstrating that cancer cells utilize autophagy as a survival mechanism in response to Abi, one of the most commonly prescribed drugs for PCa patients [14,15,16]. Consistent with our in vitro data, the main proteins involved in autophagosome formation, namely ATG12-ATG5 (ATG5) and Beclin 1, were upregulated in tumor tissue following single treatment with Abi [11]. We further show that treatment with Abi alone promoted the activation of LC3 while reducing the expression of P62 in the tumor xenograft tissue. This aligns with our and other findings, wherein we observed enhanced LC3 localization and a reduction in P62 levels following the induction of autophagy in Abi-treated cells [11,17]. In addition, we demonstrated that administering Abi alongside autophagy suppression using Chl heightens cytotoxicity in PCa cells. This combination resulted in increased apoptosis and reduced xenograft growth in castrated nude mice. Thus, the combined treatment has the potential to overcome the protective effects of autophagy on PCa cells, thereby enhancing the efficacy of Abi and its effect on promoting cell death. Research has shown that mammalian cells and tissues deficient in autophagy exhibit elevated levels of ubiquitin and P62 [18,19]. Therefore, the increase in P62 protein expression confirms that autophagy is inhibited in mice treated with Chl alone and in combination with Abi. Furthermore, this resulted in an increase in cleaved caspase 3 protein expression and a decrease in the proliferation marker Ki-67 protein, indicating enhanced apoptosis. Autophagy inhibitors like Chl are utilized to heighten the responsiveness of various cancer cells to different anticancer medications, such as cisplatin or tamoxifen, and also to radiation therapies [20,21,22]. Hence, we sought to explore the effects of Abi when combined with a substance like Chl. This FDA-approved drug is a recognized autophagy inhibitor originally designed for the prevention and treatment of malaria [23]. It inhibits autophagy during advanced stages of the autophagic process by disrupting lysosome acidification and hindering the degradation of autophagosomes [23]. Chl, whether administered alone or in conjunction with various anticancer drugs or chemotherapeutics, has been employed in numerous clinical trials for treating diverse cancers, and it has been reported to exhibit good tolerance [24,25,26]. The observed positive responses to high doses of Chl in combination with anticancer drugs in these studies support further investigation of combining anticancer drugs with Chl or more potent autophagy inhibitors. In our experimental setting, we confirmed that the combination of Chl and Abi was well tolerated, increased apoptosis, and reduced xenograft growth in castrated nude mice.

The present study supports our previous findings on the impact of apalutamide combined with the autophagy inhibitor chloroquine in a xenograft model. We demonstrated that autophagy contributes to resistance against apalutamide in vivo [13]. The combination treatment significantly enhanced the cytotoxicity of PCa cells, increased apoptosis via caspase-3 activation, and reduced tumor growth in castrated nude mice. While both drugs target androgen signaling, apalutamide directly inhibits the androgen receptor, whereas abiraterone suppresses androgen production. Apalutamide is approved for treating both non-metastatic castration-resistant PCa (nmCRPC) and metastatic castration-sensitive PCa mCSPC [27], and abiraterone, combined with ADT, effectively lowers androgen levels to slow cancer progression with significantly longer overall survival in men with newly diagnosed high-risk mCSPC [28].

Targeting key autophagic signaling pathways, such as TNK2/ACK1-mediated phosphorylation of ATP5F1A, can hinder prostate cancer progression by promoting mitophagy and reducing tumor growth [29]. Additionally, SHP2’s role in dephosphorylating histone H3 is crucial for maintaining AR homeostasis. Abiraterone has been shown to influence SHP2 activity, thereby affecting histone dephosphorylation and AR-dependent signaling events [30]. These insights highlight potential therapeutic strategies for disrupting prostate cancer progression through autophagic and epigenetic mechanisms.

Additionally, abiraterone and MDV3100 have been found to induce mitophagy in LNCaP cells, a specific type of autophagy that targets damaged mitochondria, leading to mitochondrial damage, decreased proliferation, and increased apoptosis. Mitophagy inhibition reverses these effects, promoting cell proliferation and reducing apoptosis [31].

Furthermore, the use of combination therapies, such as the dihydroorotate dehydrogenase (DHODH) inhibitor BAY2402234 with abiraterone, enhances treatment efficacy by reducing intratumoral testosterone levels, inducing apoptosis, and inhibiting tumor growth in CRPC xenografts and patient-derived xenograft organoid models [32].

Furthermore, our study investigated the relationship between autophagy, PSMA, and AR in response to treatments. We observed that in castrated mice, AR presence was exclusively seen in the vehicle control group and significantly decreased in mice treated with Abi, Chl, and the combination. After two weeks of treatments, PSMA expression was increased, but by the third week, it had decreased in all treatment groups, with a significant reduction observed particularly in the Chl treatment group. These findings underscore the immediate dynamic changes in PSMA expression in response to different treatments, correlating with AR inhibition and autophagy blockade. Modulation of autophagy may influence PSMA expression or activity in prostate cancer cells, leading to altered cell survival and tumor growth.

PSMA expression levels, which are associated with androgen signaling, can significantly influence the efficacy of PSMA-targeted therapies, positioning PSMA as a promising target for innovative prostate cancer treatments [33]. In vitro experiments have demonstrated that PCa cell lines exhibiting AR and hormonal responsive expressed PSMA, whereas PSMA expression was absent in cell lines lacking AR expression [34]. Moreover, a negative correlation was found between PSMA expression at mRNA levels and exogenous androgen stimulation, with similar inhibition observed in xenograft models in vivo [34]. Enzalutamide increased PSMA expression in cells overexpressing AR, as shown by 64Cu-J591 PET imaging, while testosterone or dihydrotestosterone caused PSMA downregulation [34]. Similarly, xenograft models demonstrated a reduction in tumor size and an elevation in PSMA expression, as observed by 68Ga-PSMA-11 PET imaging after androgen deprivation [35]. This study presented initial results from a castration-sensitive PCa patient treated with apalutamide, showing a seven-fold increase in PSMA uptake. This suggests that AR inhibition boosts PSMA expression in PCa metastases, expanding the detectable lesions in PSMA-based PET imaging [35]. Understanding the relationship between PSMA expression, AR, and autophagy inhibition could have potential therapeutic implications. Combining PSMA-targeted therapies with autophagy inhibitors or modulators may enhance the efficacy of treatment in prostate cancer.

## 5. Conclusions

This study suggests that targeting autophagy alongside Abi treatment enhances its tumor-suppressive impact in CRPC. Autophagy plays a crucial role in relieving treatment-induced stress and promoting tumor cell survival, potentially leading to resistance to antiandrogen therapies. Combining Abi with autophagy modulators like Chloroquine could be a promising approach to overcome these resistance mechanisms. Additionally, our findings indicate a potential interplay between PSMA expression and autophagy in prostate cancer cells, suggesting that combining PSMA-targeted therapies with autophagy inhibitors or modulators may improve treatment efficacy and patient outcomes.

## Data Availability

All data that support the findings of this study are available from the corresponding authors upon reasonable request.

## Supplementary Materials

The following supporting information can be downloaded at: https://www.mdpi.com/article/10.3390/cancers16193261/s1, Figure S1: Immunofluorescent quantification LC3 staining of tumor sections after 3 weeks of treatments. Data are shown as SEM of 15 measurements. Figure S2: Immunofluorescent quantification of cleaved caspase 3 staining of tumor sections after 3 weeks of treatments. Data are shown as SEM of 15 measurements. Statistical analysis was performed using the ANOVA test. **** *p* < 0.0001. Figure S3. Representative original images of automated Western blots. GAPDH (41 kDa), ATG5 (ATG5-ATG12, 58 kDa), Beclin 1 (58 kDa), and P62 (62 kDa) protein expressions were quantified in Figure 4B. Figure S4: Representative original images of automated Western blots. GAPDH (41 kDa), AR (115 kDa), and PSMA (135 kDa) protein expressions were quantified in Figure 5B.

## Abbreviations

Abi	Abiraterone
AR	Androgen receptor
ATG5	Autophagy related 5 protein
Chl	Chloroquine
CRPC	Castration-resistant prostate cancer
CY3	Cyanine-3
DAPI	4′,6-diamidino-2-phenylindole
FITC	Fluorescein isothiocyanate
PC	Prostate cancer
i.p.	Intraperitoneal
