# Supplementary material for: Simultaneous Autophagy and Androgen Receptor Inhibition in a Prostate Cancer Xenograft Model
Source: Cancers (Basel). 2024 Sep 25;16(19):3261. doi: 10.3390/cancers16193261 (PMC11482627; doi:10.3390/cancers16193261)
Supplement: Supplementary file 1 [file cancers-16-03261-s001.zip › cancers-3116842-supplementary.pdf]

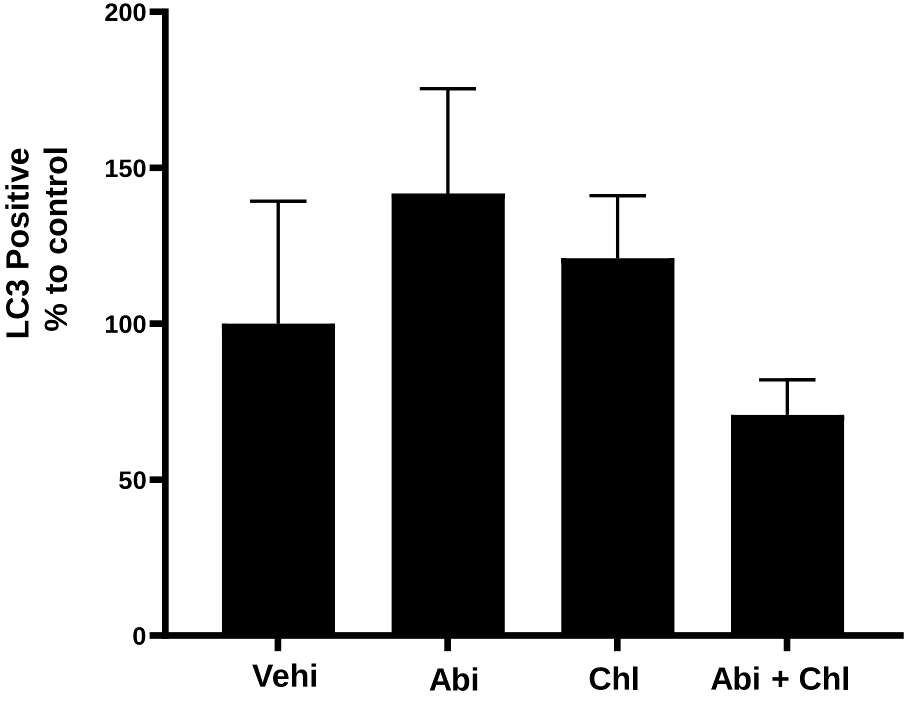

Figure S1. Immunofluorescent quantification LC3 staining of tumor sections after 3 weeks of treatments. Data are shown as SEM of 15 measurements**.**

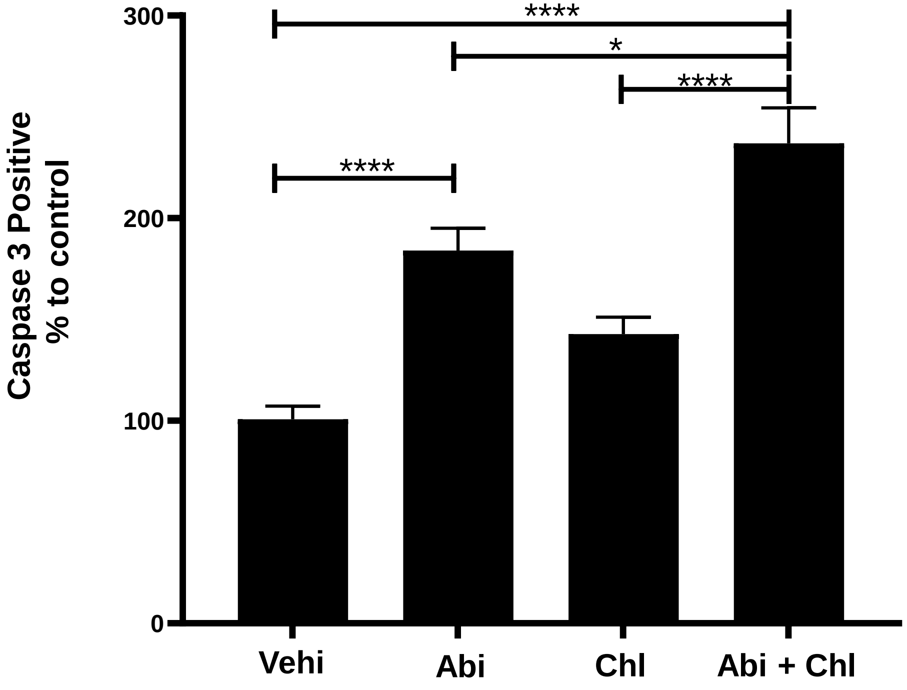

Figure S2. Immunofluorescent quantification of cleaved caspase 3 staining of tumor sections after 3 weeks of treatments. Data are shown as SEM of 15 measurements. Statistical analysis was performed using ANOVA test. ****P < 0.0001

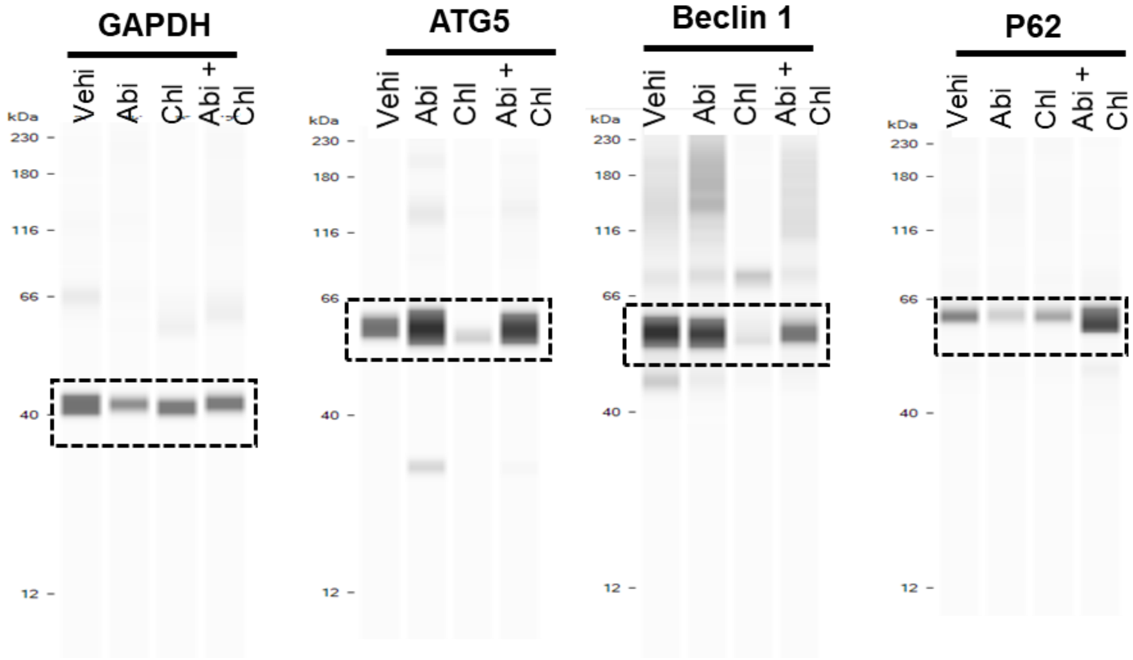

Figure S3. Representative original images of automated Western blots. GAPDH (41 kDa), ATG5 (ATG5-ATG12, 58 kDa), Beclin 1 (58 kDa), and P62 (62 kDa) protein expressions were quantified in Figure 4B.

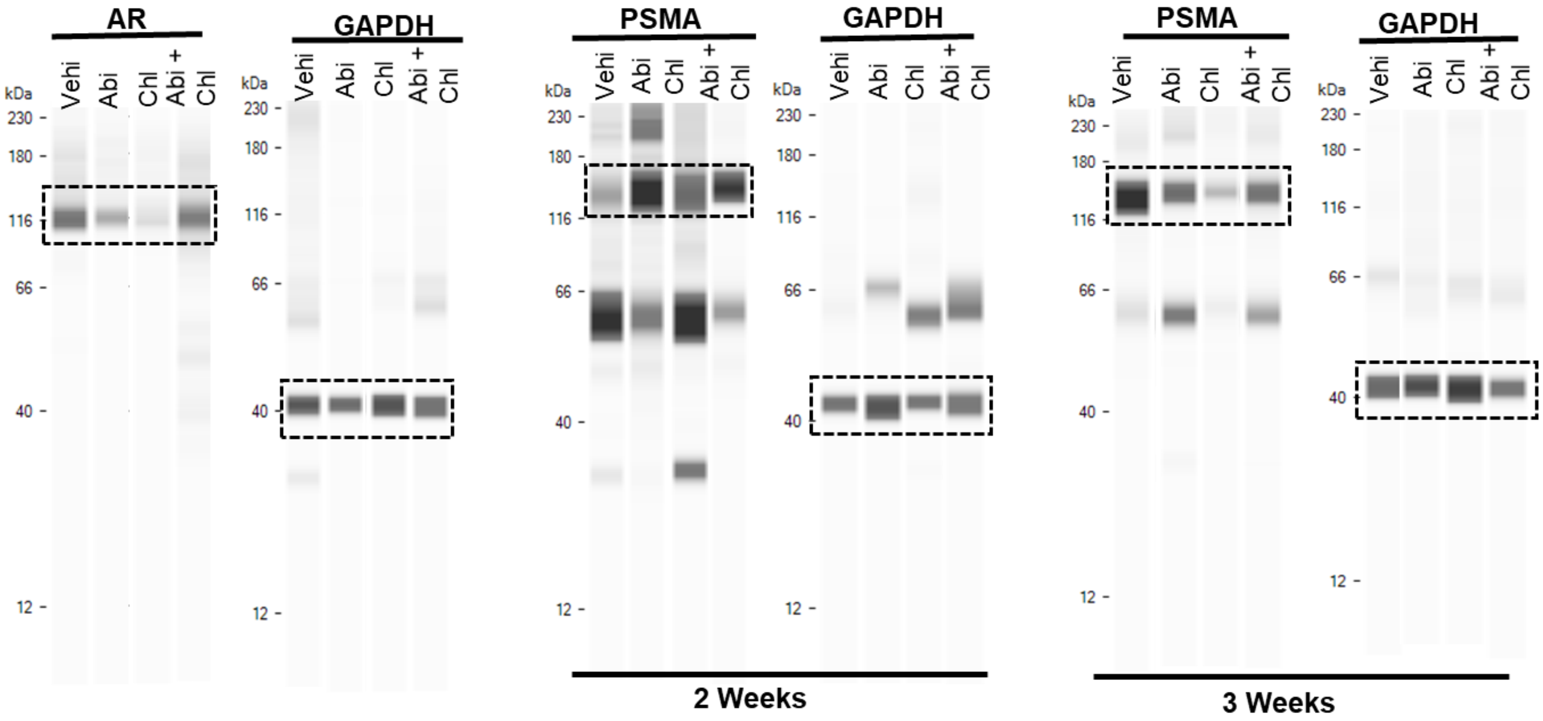

Figure S4: Representative original images of automated Western blots. GAPDH (41 kDa), AR (115 kDa), and PSMA (135 kDa) protein expressions were quantified in Figure 5B**.**
